# Reliability, validity and measurement invariance of the Simplified Medication Adherence Questionnaire (SMAQ) among HIV-positive women in Ethiopia: a quasi-experimental study

**DOI:** 10.1186/s12889-020-08585-w

**Published:** 2020-04-28

**Authors:** Chris B. Agala, Bruce J. Fried, James C. Thomas, Heidi W. Reynolds, Kristen Hassmiller Lich, Kathryn Whetten, Catherine Zimmer, Joseph P. Morrissey

**Affiliations:** 1 School of Medicine, University of North Carolina, Chapel Hill, North Carolina United States of America; 2 Health Policy & Management, UNC Gillings School of Global Public Health, Chapel Hill, North Carolina United States of America; 3 MEASURE Evaluation and Epidemiology Department, University of North Carolina, Chapel Hill, North Carolina United States of America; 4 MEASURE Evaluation, University of North Carolina, Chapel Hill, North Carolina United States of America; 5 Health Policy and Inequalities Research, Duke Global Health Institute, Duke University, Durham, North Carolina United States of America; 6 Odum Institute for Research in Social Science, University of North Carolina, Chapel Hill, North Carolina United States of America; 7 Sheps Center for Health Services Research, University of North Carolina, Chapel Hill, North Carolina United States of America

**Keywords:** HIV/AIDS care, Antiretroviral therapy, Adherence, Patient reported outcomes, Measurement invariance, Ethiopia, Sub-Saharan Africa, SMAQ, Simplified medication adherence questionnaire

## Abstract

**Background:**

Adherence to antiretroviral therapy is critical to the achievement of the third target of the UNAIDS Fast-Track Initiative goals of 2020–2030. Reliable, valid and accurate measurement of adherence are important for correct assessment of adherence and in predicting the efficacy of ART. The Simplified Medication Adherence Questionnaire is a six-item scale which assesses the perception of persons living with HIV about their adherence to ART. Despite recent widespread use, its measurement properties have yet to be carefully documented beyond the original study in Spain. The objective of this paper was to conduct internal consistency reliability, concurrent validity and measurement invariance tests for the SMAQ.

**Methods:**

HIV-positive women who were receiving ART services from 51 service providers in two sub-cities of Addis Ababa, Ethiopia completed the SMAQ in a HIV treatment referral network study between 2011 and 2012. Two cross-sections of 402 and 524 female patients of reproductive age, respectively, from the two sub-cities were randomly selected and interviewed at baseline and follow-up. We used Cronbach’s coefficient alpha (α) to assess internal consistency reliability, Pearson product-moment correlation (*r*) to assess concurrent validity and multiple-group confirmatory factor analysis to analyze factorial structure and measurement invariance of the SMAQ.

**Results:**

All participants were female with a mean age of 33; median: 34 years; range 18–45 years. Cronbach’s alphas for the six items of the SMAQ were 0.66, 0.68, 0.75 and 0.75 for T1 control, T1 intervention, T2 control, and T2 intervention groups, respectively. Pearson correlation coefficients were 0.78, 0.49, 0.52, 0.48, 0.76 and 0.80 for items 1 to 6, respectively, between T1 compared to T2. We found invariance for factor loadings, observed item intercepts and factor variances, also known as strong measurement invariance, when we compared latent adherence levels between and across patient-groups.

**Conclusions:**

Our results show that the six-item SMAQ scale has adequate reliability and validity indices for this sample, in addition to being invariant across comparison groups. The findings of this study strengthen the evidence in support of the increasing use of SMAQ by interventionists and researchers to examine, pool and compare adherence scores across groups and time periods.

## Background

According to the United Nations AIDS Program (2019), by the end of 2018 nearly 38 million people were living with HIV/AIDS, of whom 23 million were on antiretroviral therapy (ART) [1]. At the same time, 63% of the nearly 700 thousand adults living with HIV in Ethiopia were women, and new infections among young women aged 15–24 years annually were more than double those of young men, 5800 compared to 2000 [2]. HIV treatment using ART can improve functionality and decrease mortality but lapses in adherence may render treatment permanently ineffective, for example, due to drug resistance [3]. The WHO has defined adherence as “the extent to which a person’s behavior – taking medication, following a diet, and executing lifestyle changes, corresponds with agreed recommendations from a healthcare provider” [3]. Non-adherent patients have higher mortality rates than adherent ones with similar CD4+ counts and adherence is the critical determinant of survival among persons living with HIV [4–6]. Non-adherence is also associated with poor health outcomes, increased healthcare costs and poor patient safety, due to increased risk of dependence, relapses, toxicity, to mention a few [7]. Adherence is reported to be a major challenge in healthcare, estimated at 50% in high-income countries and even lower in some low and medium income countries [7]. Adherence is also critical to the achievement of the third target of the UNAIDS Fast-Track Initiative goals of 2020–2030, in which 90–95% of people with HIV are diagnosed with it, 90–95% of the diagnosed receive ART, and 90–95% of those on ART achieve viral suppression [8–10].

In Ethiopia, treatment adherence and retention were estimated to be on average 51–85% and 70% among those who had been initiated on ART, respectively [11]. In addition, a meta-analysis of 27 studies conducted in 12 sub-Saharan Africa countries (not including Ethiopia) found average adherence rates of 77% among study participants who were on ART [12]. Further, in the same meta-analysis, the authors reported average adherence of 55% among patients who participated in 24 studies in the United States and Canada [12]. In the literature, studies comparing adherence rates by sex of participants in Ethiopia are scant, but Molla et al. (2018) found that women had 1.22 higher odds of adherence to ART than men [13].

Accurate measurement of adherence is important for correct assessment of health outcomes and in predicting the efficacy of ART [7]. Non-adherence compromises treatment efficacy, and without accurate treatment efficacy data, adherence rates necessary for planning and evaluation cannot be achieved [7]. Further, accurate measurement of adherence is required for effective and efficient treatment planning, and for ensuring that changes in health outcomes can be attributed to recommended regimens. In addition, decisions to change recommendations, medications, and communication style to promote patient participation depend on valid and reliable measurement of the adherence construct [7].

Medication adherence has been measured using several methods, including: direct measures, measures involving secondary database analysis, measures involving electronic medication packaging (EMP) devices, pill count and measures involving clinician assessments and self-report [14]. However, there is no “gold standard” for measurement of adherence, and each method has advantages and disadvantages [14, 15]. For example, the WHO reported that there are challenges in measurement of the adherence construct even when more objective methods are used [7]. The report cited challenges including:

- counting inaccuracies using the “remaining dosage units” method;
- the inability to capture important information such as timing of dosage and pattern of missed dosage;
- the high cost of medication event monitoring systems (MEMS);
- the inability to tell whether patients actually use their medicine when they are removed from the bottle;
- difficulties faced when an individual acquires medication at multiple pharmacies; and
- inaccurate and incomplete records using the prescription refills method [7].

Self-reports include measures such as patient-kept diaries, patient interviews and questionnaires and scales; they tend to overestimate adherence behavior compared with other methods [15]. Despite their limitations, self-reports can significantly predict clinical outcomes and produce actionable information for patients and providers [15]. They are also cheaper, noninvasive and easier to administer compared with other methods [15]. Some examples of self-report questionnaires and scales for general use include: Adherence Estimator, Adherence to Refills and Medication Scale (ARMS), Brief Medication Questionnaire (BMQ), Medical Outcomes Study (MOS), Medication Adherence Scale (MAS), Medication Management Instrument for Deficiencies in the Elderly (MedMaIDE), Medical Adherence Measure, Morisky Adherence Questionnaire 4 item (MAQ) and the Morisky Adherence Questionnaire 8 item (MAQ) [14–16]. In addition, there are self-report questionnaires and scales specific to measurement of adherence to ART, for example: AIDS Clinical Trials Group (ACTG) Adherence Questionnaire, Community Programs for Clinical Research on AIDS (CPCRA), Antiretroviral Medication Self-Report, Self-Rating Scale Item (SRSI), Self-Reported Adherence (SERAD) Questionnaire, Self-Reported Questionnaire Assessing Adherence to Antiretroviral Medication, Simplified Medication Adherence Questionnaire (SMAQ), Visual Analog Scale (VAS), among others [14–16].

There is a dearth of literature on use of standardized scales to measure medication adherence among people on ART in Ethiopia. A systematic review of 15 ART adherence studies in Ethiopia reported that 60% of the studies used self-reports, other methods included: caregiver reports, unannounced pill counts, pharmacy refill record, medication event monitoring systems, viral load measurement, CD4 count and record review [17]. Some studies have reported challenges associated with various methods of assessing adherence among users of ART in Ethiopia. Biressaw et al. (2013) found a discrepancy in adherence levels estimated by caregiver reports and unannounced home-based pill counts. They found adherence estimated from unannounced pill count was unacceptably low, but comparable to that of Medication Event Monitoring System reported by other studies [18]. Amberbir et al. (2008) and Markos, Worku & Davey (2008) also reported using self-reports to assess adherence to ART among HIV positive individuals in Ethiopia. Both studies reported that self-reports overstated adherence levels compared to unannounced pill count due to social desirability bias, in addition to being susceptible to recall bias [19, 20]. The authors reported that despite their limitations, self-reports and pill count are widely used in Ethiopia because they are cheaper and easy to implement [17]. Self-reports have also been found to correlate with viral load and clinical outcomes [17].

The SMAQ is one of the self-report questionnaires which is increasingly used globally to assess adherence to ART and non-HIV-related medications [21]. It was developed and validated among a sample of predominantly male (72%) HIV-positive individuals in Spain, with 72% sensitivity, 91% specificity, and a likelihood ratio of 7.9 in identifying nonadherent patients as compared to medication event monitoring systems, the authors concluded that the SMAQ was reliable and valid for assessment of adherence among HIV-infected patients in most settings [21]. It has been used to assess adherence to ART in at least 12 countries, including South Africa and Kenya, in at least 25 studies and interventions between 2002 and 2018 [22–31, 20–25]. It has also been used to assess adherence to non-HIV medication in at least eight countries and 12 studies [32–41].

According to the WHO, standardized multi-item scales such as SMAQ that assess specific behaviors relating to medication recommendations may be better predictors of adherence than simple yes/no responses [7]. The underlying logic is that each indicator when used on its own may be insufficient to capture the construct, but when these indicators are combined, they represent a valid composite measure of the underlying construct of interest [42]. While standardized scales have potential advantages in understanding perceptions about adherence, literature assessing psychometric properties including reliability, validity and measurement invariance (MI) of different scales in diverse settings is sparse. In addition, standardized scales are often used with populations that may be quite different from the one in which the scales were originally validated [42]. Also, there is a natural desire to make group comparisons and conclusions about effects of interventions on the mean scale scores of expected patient outcomes [43]. However, such comparisons are justified only to the extent that these comparisons approximate differences of means on the theoretical true score of the relevant constructs, and when the means are generated from data collected using questionnaires and scales exhibiting acceptable levels of reliability and validity [15, 43]. Further, even when standardized scales are used, inferences and conclusions about observed mean differences are dependent on the between–group equivalence of the underlying measurement model [43]. However, an investigator’s ability to assess true differences between groups or across time can be hindered by measurement errors, which can limit the ability to make accurate meaningful comparisons when determining program impacts [42].

Measurement invariance is a statistical criterion that is used to assess the extent to which a standardized scale measures the same construct in each group and at each time point studied [43]. It provides a way to assess whether respondents interpreted measures conceptually similarly across groups and time and whether participation in an intervention altered the conceptual frame of reference against which a group responded to an indicator over time [42]. Measurement invariance requires that any two persons with the same level of the latent construct should obtain the same expected score on the indicators used to measure the underlying construct, regardless of the group they are in [44]. Assessment of MI helps in determining if a scale functions equivalently for all groups defined by factors such as gender, age, education, mother tongue, socioeconomic status, regional background, among others [44]. Demonstrating that a scale has MI allows an investigator to make valid comparison of construct scores such as means that yield meaningful interpretations and substantive inferences [45].

To improve clinical research on ART adherence in this population, properties of measuring instruments, such as reliability, validity, and MI must be analyzed. While the importance of reliability and validity for assessing a self-report instrument is well-understood, measurement invariance is increasingly being evaluated for valid comparisons of levels of latent outcomes to be made. Despite increasing frequency of use of the SMAQ in assessment of adherence to antiretroviral therapy, to date no study has assessed its MI and other psychometric properties such as reliability and validity in sub-Saharan Africa. Using data from a pre-post quasi-experimental evaluation study of a HIV/AIDS intervention among HIV-positive women of reproductive age in Ethiopia, hereinafter referred to as the parent study (pre refers to before intervention assessment or T1, whereas post refers to post intervention assessment or T2) [46, 47], this paper assesses the internal consistency reliability, concurrent and factorial validity, and MI for the SMAQ in this setting. These analyses build upon the parent study and add to the sparse literature about the validity of SMAQ as a HIV/AIDS treatment adherence measure.

## Methods

### Parent study

Data for this paper were obtained from a parent study conducted by MEASURE Evaluation that was funded by the United States Agency for International Development between March 2011 and December 2012. MEASURE Evaluation conducts studies globally on innovative public health interventions that have high potential positive impact on target (sub)populations and present high potential returns on investment. The parent study sought to assess the effect of a quasi-experimental organizational network intervention on HIV testing, ART initiation and adherence. The intervention was an organizational referral network improvement initiative to increase access to and quality of health services. The study followed the treatment and referral experiences of 926 HIV-positive women 18–49 years of age who were receiving ART and other healthcare services from provider agencies in an intervention or control site. Additional details of the parent study are reported in Appendix 1, and its findings have been published elsewhere [46, 47].

### Client interviews

The MEASURE Evaluation team enrolled clients, using random selection, from one large home-based care service provider that operated in both sites [46, 47]. Following a quasi-experimental design strategy, HIV-positive women were interviewed in two cross-sections, one (T1) prior to and the other (T2) 18 months following the network intervention. At T1, 402 clients were interviewed: 210 at the intervention site and 192 from the control site; at T2, 524 clients were interviewed: 268 from the intervention site and 256 from the control site. At both times, after voluntary verbal consent, clients were asked about personal and household-level demographic characteristics, HIV treatment status and medication adherence. Although some clients may have been interviewed at both T1 and T2, participation in T2 interviews was not conditional on T1 participation. There was no way to know whether a participant in T2 also participated in T1, because at both times the research team randomly sampled clients from agency caseload lists. Consequently, we analyzed the samples as if they were independent of each other. For demographic characteristics of participants: we used one-way ANOVA with Bonferroni correction to compare mean age; chi square test to compare response categories of levels of education, marital status and SMAQ items; and Kruskal Wallis test to compare mean income per week across groups. In addition, we used pairwise correlation to assess correlations between demographic variables and items of the SMAQ. The parent study was reviewed and approved by the Office of Human Research Ethics at the University of North Carolina at Chapel Hill IRB Number 11–0282, the Office of Research Ethics at FHI 360 and the Addis Ababa City Administration Health Bureau in Ethiopia [46, 47].

### Measurement of ART adherence

To assess adherence, participants who reported using ART were asked six questions from a standardized scale known as the Simplified Medication Adherence Questionnaire (SMAQ) [21], as follows: “1. Do you ever forget to take your medicine?”, “2. Are you careless at times about taking your medicine? “3. Sometimes if you feel worse, do you stop taking your medicines? “4. Thinking about the last week, how often have you not taken your medicine?” “5. Did you not take any of your medicine over the past weekend? And, “6. Over the past three months, how many days have you not taken any medicine at all? Adherence was scored as a “no” response to questions 1, 2, 3 and 5, zero response for question 4 and any response less than 2 for question 6. The six questions/items constituted the unidimensional model for measurement of adherence. The six questions assess three components of adherence to ART: intentional (question three), unintentional (questions one and two) and frequency or quantity (questions 4, 5 and 6). Intentional non-adherence refers to when a patient deliberately decides not to take their medication because of various reasons, for example feeling worse. Whereas unintentional non-adherence occurs when a patient wishes to adhere to medication but is prevented by some reason, for example, forgetfulness [48]. Questions four to six assess various aspects of frequency of non-adherence. An experienced Amharic-English speaker translated the questionnaire and then it was back-translated by an Ethiopian survey coordinator.

### Reliability and validity

Prior to conducting MI tests, we assessed SMAQ’s reliability and validity in an Ethiopian context. Reliability denotes the ability of a scale to produce consistent results when completed under similar conditions, whereas validity denotes the extent to which a scale measures the construct it is supposed to. Reliability is analogous to the scale’s precision, whereas validity is analogous to its accuracy.

### Internal consistency reliability and concurrent and factorial validity

We conducted an internal consistency reliability test of the SMAQ data from Ethiopia using Cronbach’s alpha (α). This index measures internal consistency reliability of both items and the construct being measured [49, 50]; in this case, how closely-related the six items of the SMAQ were as a set in measuring the adherence construct. Values of α in the range of 0.6 to 0.8 (0.6 ≤ α ≤ 0.8) are considered adequate, while 0.8 or higher is considered a high value of internal consistency [51]. We used Pearson product-moment correlation coefficients (*r*) to assess concurrent validity of the domain scores at T1 and T2. In this context, concurrent validity represents the extent to which item scores at T1 related to those of the same scale administered to women at T2 [52]. Criteria for concurrent validity were based on directionality of expected relationships of the six items between the two times and strength of the observed correlation coefficient. The Pearson product-moment correlation coefficient has a range of − 1 to + 1 between two sets of scores, and coefficients close to 1 in absolute value indicate high concurrent validity [52]. Based on thresholds from previous studies, correlation coefficients less than or equal to 0.25 suggest a weak relationship, those between 0.25 and 0.50, a moderate relationship, those between 0.50 and 0.75, strong relationship; and values greater than 0.75, very strong correlation [53, 54]. Finally, we used model fit indices and statistical significance of factor loadings to assess factorial validity. Factorial validity is one of the different, but inter-related elements of construct validity. A strong correlation between a set of indicators and a latent construct indicates factorial validity [55].

### Measurement model

**Fig. 1:**
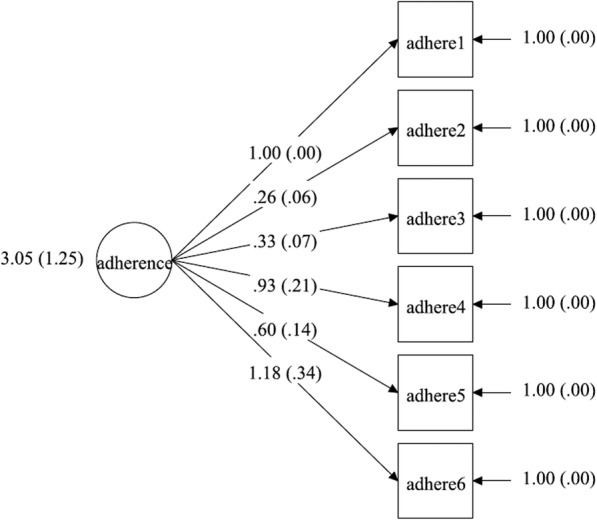
Unidimensional measurement model for adherence with variance (s.e.) of adherence score, factor loadings and measurement errors for T1 control group

**Fig. 2:**
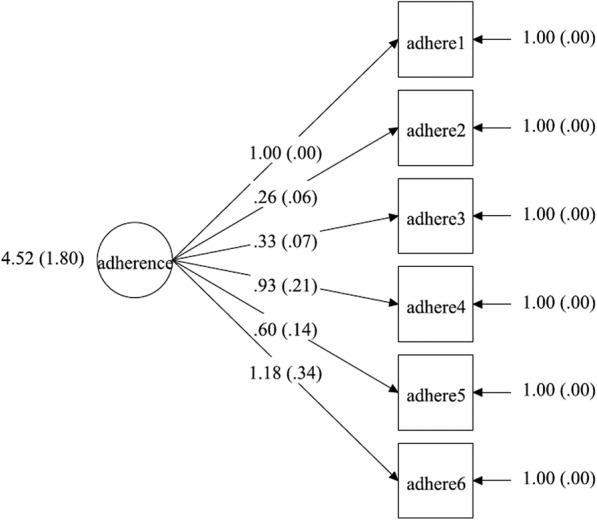
Unidimensional measurement model for adherence with mean variance (s.e.) of score, factor loadings and measurement errors for T1 intervention group

**Fig. 3:**
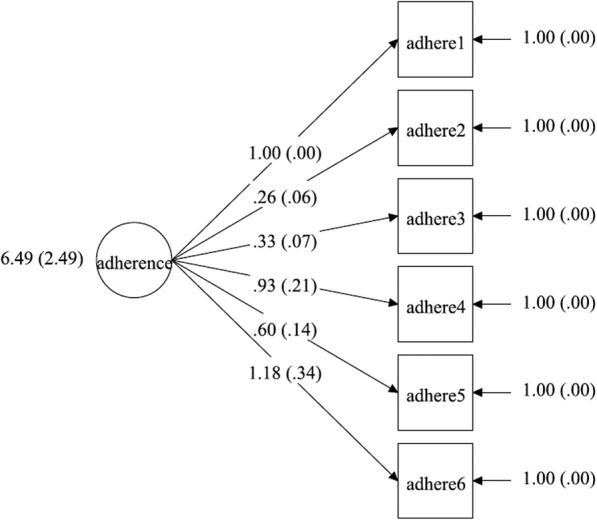
Unidimensional measurement model for adherence with variance (s.e.) of adherence score, factor loadings and measurement errors for T2 control group

**Fig. 4:**
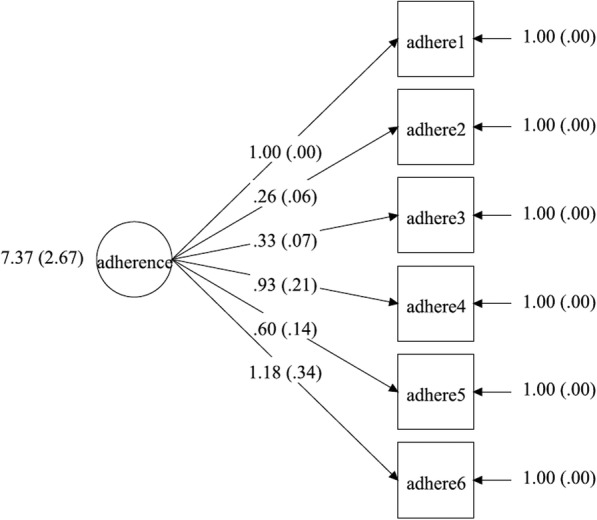
Unidimensional measurement model for adherence with variance (s.e.) of adherence score, factor loadings and measurement errors for T2 intervention group

We measured adherence with six factor indicators corresponding to the six SMAQ items. In Figs. 1, 2, 3, 4, the latent factor of adherence is represented by the circular shape. The arrows represent factor loadings, which are direct effects of each adherence indicator on the latent construct of adherence. We report summary statistics, factor loadings and model fit indices for specific models including chi-square values, root mean square error of approximation (RMSEA) values, comparative fit indices (CFI)/Tucker-Lewis indices and the final estimated measurement models. A significant chi-square test indicates a poor model fit, but this may also be due to moderate discrepancies in normality of data and large (*n* > 200) sample size [56]. Therefore, we used other model fit indices to supplement the chi-square test in determining the model that best fit the data. The RMSEA is a measure of the estimated discrepancy between the population and model implied covariance matrices per degree of freedom [43]. Values of RMSEA less than 0.05 indicate close model fit whereas values up to 0.08 reflect adequate fit. The CFI varies from 0 to 1, representing extremely weak to perfect fit, respectively, and a value of 0.95 is considered to represent adequate fit [43].

### Measurement invariance test

Measurement invariance testing is based on the overall assumption that comparison between groups is important, and the presence or absence of differences between groups has some meaningful implications [45]. We tested for the levels of invariance based upon the following assumptions: (1) the measure of interest, that is adherence to ART, was perceptually based; adherence comprises multiple manifest indicators (i.e., multiple items of the SMAQ); (2) the six items of SMAQ are combined additively to operationalize the underlying construct; (3) evidence exists of the SMAQ’s psychometric soundness beyond the preliminary stages of scale development, i.e. evidence exists of the SMAQ’s psychometric soundness in a Spanish sample, but it has yet to be demonstrated for Ethiopia or other sub-Saharan Africa location; (4) the four study groups are independent of each other: T1 control, T1 intervention, T2 control and T2 intervention; and (5) the common factor model for describing relationships among items of the SMAQ holds across groups [45].

Following the independent groups assumption, we applied a multiple-group confirmatory factor analysis (CFA) to test three levels of invariance: configural, weak factorial and strong factorial [45]. Multiple-group CFA allowed us to simultaneously test four group-specific latent adherence factor models using robust weighted least squares (WLSMV). We fit models for each group/time and evaluated sample differences with a chi-square test. We used WLSMV to conduct chi-square difference testing because adherence indicators were categorical and non-normally distributed. A significant chi-square difference value indicated that constraining the parameters of the nested model significantly worsened the fit of the model, which indicated measurement non-invariance, thereby sustaining the unconstrained or less constrained model. A non-significant chi-square difference indicated that constraining the parameters of the nested model did not significantly worsen the fit of the model, which indicated MI of the parameters constrained to be equal in the nested model. We did not estimate the next restrictive model if the result was significant, as it suggested that the next level of parameter restriction would have significant differences with the previous model. We used MPlus 7 [57] and Stata 12 [58] to conduct data analysis. Additional details of steps for invariance testing can be found in Appendix 2.

## Results

Study participants were 926 female clients who were 18–45 years of age and receiving HIV care from a home-based care organization in each sub-city. Their overall mean age was 33 (across the four groups 33.06–33.74; median: 34 years). Participants in the two intervention groups had significantly higher mean age (34 years) compared to Control group (32 years) at T1 (*F*(3, 922) = 4.67, *p* < 0.01). Significantly higher proportion of participants were married across all groups *X*^*2*^*(*df = 3) = 8.70, *p* = 0.03), with only one third of the participants living with their sexual partner across groups. There were significant differences in categories of levels of education across study groups (*X*^*2*^*(*df = 21) = 60.66, *p* < 0.01), with nearly one quarter of all participants had no formal education, and only 15% had post-primary education. There were significant differences in mean weekly income across groups (*X*^*2*^*(*df = 3) = 143.24, *p* < 0.01), and of the 80% who reported their weekly income, the average income was 2011 US$4 (range US$ 0–72) (Table 1).

**Table 1:** Demographic characteristics of 926 HIV-positive women who participated in reliability and validity testing of the Simplified Medication Adherence Questionnaire (SMAQ) by study group

Variable	Control T1	Intervention T1	Control T2	Intervention T2	*p*-value
Age, mean (SD)	32(6)	34(6)	34(5)	34(6)	< 0.01
Education					
1) No school	33%	16%	29%	17%	< 0.01
2) Adult education	5%	5%	7%	8%	
3) Primary (1–4 grades)	17%	20%	22%	17%	
4) Primary (5–8 grades)	34%	39%	29%	40%	
5) Secondary (9–10 grades)	6%	13%	11%	17%	
6) Preparatory (11–12 grades)	5%	6%	1%	2%	
7) Technical/vocational certificate	0	0.5%	0	0	
8) University degree/associate degree	0.5%	0.5%	0	0	
Marital status					
1) Married	40%	26%	33%	31%	0.03
2) Not Married	60%	74%	67%	69%	
Income per week in US$, mean (SD)^a^	3(7)	4(7)	5(6)	6(6)	< 0.01
N	192	210	256	268	

^a^Exchange rate: 2011 US$ 1 = Ethiopian Birr 17.2836 (Source: National Bank of Ethiopia)

### Correlations between adherence measurement items of the SMAQ

Participant responses to the six SMAQ items/questions on adherence are presented in Table 2. Chi square tests showed no differences in response categories of the SMAQ across the four groups (X^2^(df = 3) = 0.58–6.64, *p >* 0.05). Initial assessment of correlations between the six items ranged from − 0.09 to 0.95 (see Table 3). Question five “did you not take any of your medicine over the last weekend” was not significantly correlated (correlation coefficient = − 0.09), with question three “sometimes if you feel worse, do you stop taking your medicines?” in the T1 intervention group. This was not expected, as all indicators of a construct are expected to have significant positive correlations with each other. Due to the negative correlation coefficient and its nonsignificance, we considered removing question five from our analysis, but sensitivity analysis with and without this item showed no differences in results for measurement invariance tests. Therefore, we included it in order to present results for the full SMAQ scale as it was originally designed and validated in Spain. Further, the demographic variables had substantially weak correlation with SMAQ items, and thus we did not include demographic variables in the multiple group confirmatory factor analysis (0.05 < r < 0.105).

**Table 2:** Patient responses to the six-item Simplified Medication Adherence Questionnaire (SMAQ) at baseline (T1) and follow-up (T2) in the intervention and control groups of the Ethiopian HIV/AIDS parent study

Question	T1 Responses to adherence questions (%)		T2 Responses to adherence questions (%)		(X^2^(df) *p*-value)
	Control: *n* = 192	Intervention: *n* = 209	Control: *n* = 256	Intervention: *n* = 265	
Do you ever forget to take your medicine?	Yes 60 (31)	Yes 76 (36)	Yes 66 (26)	Yes 77 (29)	(X^2^(df = 3) = 6.39 *p =* 0.09
	No 132 (69)	No 133 (64)	No 190 (74)	No 188 (71)	
Are you careless at times about taking your medicine?	Yes 23 (12)	Yes 31 (15)	Yes 21 (8)	Yes 30 (11)	(X^2^(df = 3) = 4.97 *p =* 0.17
	No 169 (88)	No 178 (85)	No 233 (92)	No 235 (89)	
Sometimes if you feel worse, do you stop taking your medicines?	Yes 14 (7)	Yes 19 (9)	Yes 21 (8)	Yes 24 (9)	(X^2^(df = 3) = 0.58 *p =* 0.90
	No 178 (93)	No 190 (91)	No 233 (92)	No 241 (91)	
Thinking about the last week. How often have you not taken your medicine?	Never 162 (84)	Never 169 (81)	Never 222 (87)	Never 223 (84)	(X^2^(df = 3) = 2.97 *p =* 0.40
	1–2 times 22 (11)	1–2 times 33 (16)	1–2 times 27 (11)	1–2 times 33 (12)	
	3–5 times 6 (3)	3–5 times 7 (3)	3–5 times 5 (2)	3–5 times 7 (3)	
	> 5 times 2 (1)	> 5 times 0 (0)	> 5 times 2 (1)	> 5 times 2 (1)	
Did you not take any of your medicine over the past weekend?	Yes 9 (5)	Yes 15 (7)	Yes 13 (5)	Yes 11 (5)	(X^2^(df = 3) = 2.33 *p =* 0.51
	No 178 (95)	No 194 (93)	No 235 (95)	No 254 (95)	
Over the past 3 months, how many days have you not taken any medicine at all?	≤ 2 days 183 (95)	≤ 2 days 189 (90)	≤ 2 days 227 (89)	≤ 2 days 244 (92)	(X^2^(df = 3*)* = 6.64 *p =* 0.08
	> 2 days 9 (5)	> 2 days 20 (10)	> 2 days 28 (11)	> 2 days 21 (8)

**Table 3:** Correlation matrices for participant responses to the six items/questions of the Simplified Medication Adherence Questionnaire (SMAQ)

a. Correlation matrices for the responses to the six adherence indicators for T1 control group							c. Correlation matrices for the responses to the six adherence indicators for T2 control group						
	Adhere1	Adhere2	Adhere3	Adhere4	Adhere5	Adhere6		Adhere1	Adhere2	Adhere3	Adhere4	Adhere5	Adhere6
Adhere1	1.00						Adhere1	1.00					
Adhere2	0.20	1.00					Adhere2	0.56	1.00				
Adhere3	0.43	0.62	1.00				Adhere3	0.52	0.66	1.00			
Adhere4	0.73	0.04	0.35	1.00			Adhere4	0.85	0.62	0.65	1.00		
Adhere5	0.55	0.20	0.35	0.75	1.00		Adhere5	0.49	0.38	0.49	0.53	1.00	
Adhere6	0.75	0.31	0.51	0.70	0.70	1.00	Adhere6	0.88	0.56	0.53	0.86	0.68	1.00
b. Correlation matrices for the responses to the six adherence indicators for T1 intervention group							d. Correlation matrices for the responses to the six adherence indicators for T2 intervention group						
	Adhere1	Adhere2	Adhere3	Adhere4	Adhere5	Adhere6		Adhere1	Adhere2	Adhere3	Adhere4	Adhere5	Adhere6
Adhere1	1.00						Adhere1	1.00					
Adhere2	0.30	1.00					Adhere2	0.46	1.00				
Adhere3	0.44	0.24	1.00				Adhere3	0.62	0.68	1.00			
Adhere4	0.80	0.21	0.31	1.00			Adhere4	0.85	0.46	0.56	1.00		
Adhere5	0.65	0.11	−0.09	0.92	1.00		Adhere5	0.51	0.29	0.36	0.69	1.00	
Adhere6	0.71	0.55	0.51	0.73	0.58	1.00	Adhere6	0.95	0.42	0.63	0.87	0.64	1.00

### Internal consistency reliability and concurrent and factorial validity

The six items of the SMAQ exhibited adequate or moderately strong internal consistency reliability in measuring the latent construct of adherence at T1, T2 and in the full sample. The Cronbach’s α was 0.66 and 0.68 at T1 for control and intervention groups respectively; 0.75 at T2 for both groups and 0.72 for the full sample. Concurrent validity of the six-item SMAQ exhibited moderate to excellent positive correlations for the full sample between T1 and T2. Pearson correlation coefficients of items 1, 5 and 6 were excellent for T1 compared to T2 (item 1 = 0.78, item 5 = 0.76, item 6 = 0.80); similarly, correlations of item 3 was good (item 3 = 0.52) and that for item 2 and item 4 were moderate (item 2 = 0.49, item 4 = 0.48) (see Table 4). The good model fit indices and significant factor loadings across the four groups indicate factorial validity, and that the scale performed equally well when T1 control, T1 intervention, T2 control and T2 intervention were all compared using multiple group confirmatory factor analysis. (See Tables 5 and 6).

**Table 4:** Estimates of Cronbach’s Alpha (α), Pearson’s correlation coefficient (r) for Simplified Medication Questionnaire (SMAQ) scores at T1, T2 and for overall sample

Internal consistency reliability	
Group	Cronbach’s Coefficient alpha (α)
T1 control	0.66
T1 intervention	0.68
T2 control	0.75
T2 intervention	0.75
All groups	0.72
Concurrent validity for the full sample	
SMAQ item	Pearson’s Correlation (r)
Do you ever forget to take your medicine?	0.78
Are you careless at times about taking your medicine?	0.49
Sometimes if you feel worse, do you stop taking your medicines?	0.52
Thinking about the last week. How often have you not taken your medicine?	0.48
Did you not take any of your medicine over the past weekend?	0.76
Over the past 3 months, how many days have you not taken any medicine at all?	0.80

**Table 5:** Model fit indices and measurement invariance test for the six-item Simplified Medication Adherence Questionnaire (SMAQ) among female HIV/AIDS clients in Ethiopia

Model fit indices	Configural invariance model		Weak factorial invariance model		Strong invariance model	
	Estimate	*p*-value	Estimate	*p*-value	Estimate	*p*-value
Chi-square (degree of freedom)	76.28 (36)	0.00	106.85 (51)	0.00	122.82 (66)	0.00
Root mean square error of approximation (RMSEA) (90% Confidence interval)	0.07 (0.05–0.09)	0.07	0.07 (0.05–0.09)	0.04	0.06 (0.04–0.08)	0.13
Comparative Fit Index (CFI)	0.99		0.99		0.99	
Tucker Lewis Index (TLI)	0.98		0.98		0.99	
Chi-square difference (degrees of freedom)	–		34.79 (15)	0.00	13.36 (15)	0.57

**Table 6:** Multiple-group confirmatory factor analysis and measurement invariance levels of the six-item Simplified Medication Adherence Questionnaire (SMAQ) for control and intervention groups at T1 and T2 among female HIV/AIDS clients in Ethiopia

	Configural		Weak factorial		Strong factorial	
	Factor Loadings		Factor Loadings		Factor Loadings	
	Estimate	*p*-value	Estimate	*p*-value	Estimate	*p*-value
**Control group at T1**						
Do you ever forget to take your medicine?	1.00	–	1.00	–	1.00	–
Are you careless at times about taking your medicine?	0.26	0.05	0.26	0.00	0.26	0.00
Sometimes if you feel worse, do you stop taking your medicines?	0.48	0.03	0.33	0.00	0.33	0.00
Thinking about the last week. How often have you not taken your medicine?	1.00	0.02	0.97	0.00	0.93	0.00
Did you not take any of your medicine over the past weekend?	0.85	0.03	0.63	0.00	0.60	0.00
Over the past 3 months, how many days have you not taken any medicine at all?	1.15	0.02	1.12	0.00	1.18	0.00
**Intervention group at T1**						
Do you ever forget to take your medicine?	1.00	–	1.00	–	1.00	–
Are you careless at times about taking your medicine?	0.29	0.01	0.26	0.00	0.26	0.00
Sometimes if you feel worse, do you stop taking your medicines?	0.33	0.01	0.33	0.00	0.33	0.00
Thinking about the last week. How often have you not taken your medicine?	2.95	0.27	0.97	0.00	0.93	0.00
Did you not take any of your medicine over the past weekend?	1.34	0.00	0.63	0.00	0.60	0.00
Over the past 3 months, how many days have you not taken any medicine at all?	0.92	0.00	1.12	0.00	1.18	0.00
**Control group at T2**						
Do you ever forget to take your medicine?	1.00	–	1.00	–	1.00	–
Are you careless at times about taking your medicine?	0.40	0.02	0.26	0.00	0.26	0.00
Sometimes if you feel worse, do you stop taking your medicines?	0.41	0.02	0.33	0.00	0.33	0.00
Thinking about the last week. How often have you not taken your medicine?	1.00	0.01	0.97	0.00	0.93	0.00
Did you not take any of your medicine over the past weekend?	0.36	0.03	0.63	0.00	0.60	0.00
Over the past 3 months, how many days have you not taken any medicine at all?	1.31	0.09	1.12	0.00	1.18	0.00
**Intervention group at T2**						
Do you ever forget to take your medicine?	1.00	–	1.00	–	1.00	–
Are you careless at times about taking your medicine?	0.21	0.03	0.26	0.00	0.26	0.00
Sometimes if you feel worse, do you stop taking your medicines?	0.30	0.03	0.33	0.00	0.33	0.00
Thinking about the last week. How often have you not taken your medicine?	0.58	0.03	0.97	0.00	0.93	0.00
Did you not take any of your medicine over the past weekend?	0.26	0.05	0.63	0.00	0.60	0.00
Over the past 3 months, how many days have you not taken any medicine at all?	2.64	0.64	1.12	0.00	1.18	0.00

### Measurement model

Three multiple group CFA’s were conducted: for configural model (χ2 = 76.28 (degrees of freedom = 36); *p* < 0.01, CFI = 0.99; TLI = 0.98; RMSEA = 0.07), weak factorial model (χ2 = 106.85 (degrees of freedom = 51); *p* < 0.01, CFI = 0.99; TLI = 0.98; RMSEA = 0.07) and strong factorial model (χ2 = 122.82 (degrees of freedom = 66); *p* < 0.01, CFI = 0.99; TLI = 0.99; RMSEA = 0.06), separately. All the models exhibited good fit based on CFI (CFI =0.99). Model fit based on RMSEA was best in the strong factorial model (RMSEA = 0.06 (90% CI 0.04–0.08)), suggesting that constraining factor loadings, intercepts and factor variances improved model fit in the strong factorial model, compared with the configural and weak factorial models (see Table 5). Factor loadings in the final strong factorial model were all statistically significant (*p* < 0.05) and ranged from 0.26 to 1.18 (see Table 6). Positive and significant factor loadings suggest that the construct of adherence significantly and positively influenced all the measures generated by the six items of SMAQ.

### Measurement invariance test

A chi-square difference test between configural and weak factorial models was significant (chi-square difference = 34.79 (DF = 15) *p* < 0.01). Other model fit indices were comparable between the two models, which suggested that the weak factorial model had a better fit for the data. The next chi-square test between the weak and strong factorial models found no significant difference (chi-square difference = 13.36 (DF = 15) *p* = 0.57). In addition, the RMSEA statistic reduced by 0.01 to 0.06 and other model fit indices were comparable with those of the weak factorial invariance model. Therefore, the strong factorial invariance model had the best fit for the data and was accepted as the final model (see Tables 5 and 6). The final estimated measurement models for the strong factorial invariance are presented in Figs. 1, 2, 3, 4. Factor loading estimates for the models are shown in Table 6.

## Discussion

The purpose of this study was to assess the psychometric and related measurement properties of the six-item SMAQ using data from a quasi-experimental parent study of HIV-positive women of reproductive age in Ethiopia. Our findings indicate that the six-item SMAQ demonstrated adequate internal consistency reliability, suggesting that the six items in the questionnaire reflect the same latent construct of adherence to antiretroviral therapy. In addition, concurrent validity of the scale was moderate to excellent based on correlations between the item responses at T1 compared with T2. Further, model fit indices and significant factor loadings demonstrated factorial validity, which suggests construct validity as well.

In addition, we documented strong factorial invariance across the four independent study groups, suggesting that the SMAQ questions/items were being interpreted in an equivalent manner across groups. This finding suggests that the SMAQ performs equally well across samples and operationalizes group-specific differences in an invariant manner across groups. An important implication of this finding is that adherence scores obtained using SMAQ from the four study groups can be compared pre-and post-intervention for policy or intervention purposes [37]. Taken together, these findings affirm that the six-item SMAQ is a valid measure of adherence to ART in this sample of women with HIV/AIDS in Ethiopia. Our findings add confidence for researchers and interventionists interested in using the SMAQ to assess adherence to ART in this setting.

We found one negative but nonsignificant correlation between the six indicators of the SMAQ suggesting that a five-item version might be more efficient [59]. However, our findings showed no differences in measurement invariance tests when question five was included or excluded. It is possible that the lack of correlation was caused by a data entry error, but we were unable to verify this possibility. More likely, it was due to the magnitude of question five’s correlation with question three being too small to impact the results. In addition, question five was strongly and positively correlated with other items of the scale, and all its factor loadings were positive and significant. Thus, we maintained the integrity of the original six-item SMAQ scale in our final analyses.

In comparison with the validation study in Spain, the mean age of patients in the present study was slightly lower (33 years versus 36 years). All participants in our study were female compared to 28% in the Spanish study. The Cronbach’s α in the present study was lower for T1, but comparable for T2 and for the full study sample (α = 0.75) [21]. Estimating concurrent and factorial validity was a quick way to validate our SMAQ data, although predictive validity would be a more powerful criterion for future studies predicting SMAQ scores in relation to ART interventions where the counts of HIV ribonucleic acid (RNA) or CD4 T lymphocytes (CD4 cells), for example, are available.

The SMAQ has several advantages for field studies—it is short and easier to administer, which makes identification of non-adherent patients and intervening quicker at crowded health service facilities associated with severe personnel shortages and long waiting times [60]. Conversely, collection of HIV RNA or CD4 counts requires much more time, financial and workforce recourses which were limited in the study setting. Other studies have used data from two cross sections to assess validity of standardized scales [52]. Our study complemented the need for assessment of validity by testing for measurement invariance of the SMAQ and found it to be invariant across groups and time, suggesting that the six items are relevant for measurement of the latent factor of adherence to ART.

The Morisky Scale and variations of the adult AIDS Clinical Trials Group (ACTG) are also used to assess self-reports of adherence [15, 61, 62]. The SMAQ is a modified version of the original four-item Morisky Scale, which has since been modified and validated as an eight-item scale [61, 63]. However, the Morisky Scale has been validated and is more commonly used in hypertension patients and general purpose adherence studies and interventions, compared to the SMAQ and ACTG which have been validated with persons living with HIV [62, 64]. Compared to the SMAQ, the ACTG scale is longer and would require much more time and higher costs to administer and collate participant responses into actionable insights that can guide quick adherence improvement interventions. Self-reports of adherence seek various types of information from respondents, including: medication-taking behavior, and barriers and beliefs associated with adherence [64]. The choice of a self-report measure depends on the goal of the study or intervention. The SMAQ seeks information about medication-taking behavior and barriers to adherence [64]. The parent study assessed level of adherence with the goal of improving adherence by reducing ART access barriers by increasing the number of access points or service providers. In this way, the SMAQ was more appropriate to the goals of the parent study than the ACTG. Researchers and interventionists with similar needs and goals may consider using the SMAQ in their studies.

Initiation, retention and adherence to ART positively influence quality of life among persons living with HIV [65–69], are required for viral suppression, and are critical to the achievement of the UNAIDS 90–90-90 goals. However, initiation and retention on ART are only meaningful to the extent to which users of ART can adhere to the regimen [70]. Also, recent studies have shown that adherence to ART can be a successful HIV prevention strategy [8, 71, 72]. Improving adherence may be challenging or impossible without our ability to measure it reliably, validly and consistently across groups of individuals, which makes efforts to improve measurement methods and tools an important contribution for public health. A study of strategies to improve adherence to ART in low-resource settings reported that adherence measurement was required for optimal targeting and tailoring of interventions [73]. The present study moves the field forward by presenting reliability, validity and invariance test statistics for SMAQ from a sub-Saharan Africa setting where such HIV research is scant, yet the burden of disease and potential need for such measurement is greatest as sub-Saharan Africa bears the greatest HIV/AIDS burden. According to the WHO, nearly one in every 25 adults is living with HIV in Africa, accounting for nearly two-thirds of the global total [74]. Providing evidentiary measurement properties for SMAQ increases practitioners’ confidence in using SMAQ, which increases its adoption in assessment of adherence.

Although we found strong invariance for the six items of the SMAQ, it is worthwhile to note that adherence is a dynamic behavior which may change over time, even without intervention. Thus, invariance can be expected for SMAQ items that assess intentional non-adherence across time, such as question three of the SMAQ: “Sometimes if you feel worse, do you stop taking your medicines?”, because such items are embedded in a patient’s beliefs and self-construct and therefore, are more robust to behavior change. Conversely, the SMAQ also has a component of unintentional non-adherence due to forgetfulness, assessed by questions one and two: “Do you forget to take your medicine?” and “Are you careless at times about taking your medicine”? The Unintentional non-adherence component may be prone to random variability, which may not be captured by invariance testing of the six items of the SMAQ together, but by testing invariance for each item using longitudinal data. Thus, attribution of changes in adherence to specific components of the SMAQ as intentional or unintentional was not possible in the present study, because of the independent cross-section design. This is an important area for future studies in which researchers may be able to identify modifiable items of non-adherence measured by the SMAQ so as to appropriately intervene to improve adherence, as was demonstrated by Mora et al. (2011) in their assessment of non-adherence among asthma patients using the Medication Adherence Report Scale (MARS-A10) [75].

Several limitations of our study should be noted. Although we treated the samples as independent, they may not be truly independent because some participants may have participated at both T1 and T2 interviews. This limitation may manifest in repeated questions where social desirability bias is also a limitation. However, the cross-sectional design of the parent study mitigated this tendency. In addition, statistical tests showed group differences in demographic characteristics. The design limited the use of multilevel multigroup CFA, as suggested by Kim and colleagues [76]. Ethical considerations and operational logistics were also considered in the design. The taxonomy for describing adherence to medications now suggests that results from baseline and follow-up can only be compared if the patient was already on treatment at least 3 months prior to baseline [77]. However, the taxonomy was not in place at the time of data collection. Challenges associated with diagnosis and treatment initiation records in the study settings would also limit application of the taxonomy. Further, the lack of data on clinical methods of measuring adherence—such as a HIV RNA test (a test that checks for RNA genetic material from the virus in a sample of blood) [78, 79] or CD4 counts (the number of CD4 T lymphocytes – a type of white blood cells -- in a sample of blood, which is used to monitor an individual’s response to ART) [80]—limited our ability to assess the predictive validity of the SMAQ with these data. This is an important agenda for future research.

## Conclusions

This is the first study to assess reliability, validity and measurement invariance of SMAQ in the sub-Saharan Africa region, using pre- and post-intervention data from two treatment referral networks. The findings show that the SMAQ is sufficiently reliable and valid to be used for HIV-positive Ethiopian women of reproductive age who are on ART. In addition, the findings demonstrate that comparisons across groups are possible in the study sample and in future, and unlikely to be affected by differences in response styles, interpretations of indicators, time lapse and socio-economic factors [81]. Further research is warranted to determine whether the measurement properties of SMAQ reported here would hold among participants from other countries for males and females, of different age groups, from various regions, and various socio-economic statuses.

## Data Availability

The data are available at the Carolina Population Center at the University of North Carolina at Chapel Hill, North Carolina, USA, and can be obtained upon request.

## Appendix 1

### Details of the methodology of parent study by MEASURE Evaluation researchers

Data for the present study are from a quasi-experimental referral network study conducted by MEASURE Evaluation (https://www.measureevaluation.org). Kirkos was the site of an intervention aimed at improving ties among providers. Providers were identified using snowball sampling, beginning with well-known service providers as the seeds. All service providers that offered, and could refer clients to one another for, HIV care and treatment support and family planning services to HIV-positive women of ages 18–49 were included. Saturation was reached when nominated organizational representatives, who were also the interviewees for the study, named service providers that had already been named by others. Ultimately, 25 providers.

in Kirkos and 26 in Kolfe-Keranyo were included in the study. The data included provider characteristics.

and linkages among providers. To obtain referral network data, T1 (baseline) and T2 (follow-up) interviews were conducted with nominated key informants in each provider organization about HIV services and the nature and types of referrals offered, provider characteristics, collaborations, joint programs, and linkages with other providers.

### The intervention

A referral network strengthening intervention was implemented in Kirkos where 21 of the 25 providers were represented in at least one of the meetings. Kirkos was selected for the intervention after T1 results showed lower network density there compared with Kolfe-Keranyo. The intervention consisted of a series of three 2-day educational meetings held 2 months apart at different times after T1 data collection. During the meetings, participants learned about strategies for client referral, collaboration, joint programming, and partnerships. They also learned about services offered by other facilities in the network. Service directories, listing contact information and services offered, were developed and shared, with each participating provider receiving at least one directory. No intervention was implemented in Kolfe-Keranyo, the control network. Characteristics of service providers Service providers in the two networks were owned and operated by the Ethiopian government or various types of non-governmental bodies. The government owned and operated 10 out of the 51 service providers, whereas non-governmental organizations (NGOs), faith-based providers (FBOs) or private individuals owned and operated the remainder. Kirkos had significantly fewer (three) government owned and operated providers compared with Kolfe-Keranyo, which had seven. There was a significant increase in the number of providers self-identifying as NGOs in Kirkos from five at T1 to 14 at T2. Conversely, Kolfe-Keranyo experienced a reduction in the number of NGOs from eight at T1 to five at T2.

## Appendix 2

### Invariance testing

An invariant measurement model has equal factor loadings, intercepts, factor variances and residual variances across groups. We applied MI testing methods described by Bollen (1989) and Widaman & Reise (1997) [82, 83]. The most basic form of invariance is configural or pattern invariance, followed by weak, strong and strict factorial invariance, in that order. Measurement invariance was assessed by testing the invariance of measurement parameters including: factor loadings, intercepts and factor variances across the four groups [82, 83]. We tested for MI by consecutively imposing additional constraints on successive levels of invariance. For configural invariance test, we conducted independent group-specific CFAs with no modifications to assess overall fit of the model within each study group. Next, we equated factor loadings and fixed mean adherence to zero to test for weak factorial. A factor loading is the strength of the linear relation between a latent factor and each of the six items of the scale [43, 82]. Finally, we tested for strong invariance by fixing factor means for adherence in one group and constraining factor loadings, intercepts and factor variances to be equal, whereas residual variances were freely estimated. Strong invariance justifies across time and between group comparisons [42, 43, 82]. We did not test for strict invariance because residual variances would vary from group to group even if all the groups were from one population.

### Levels of invariance

1. **Configural invariance**

Under configural invariance, we were interested in testing whether the same indicators measured the construct of interest across multiple groups [42]. The model structure requires that the same item must be an indicator of the latent factor in each group, but the magnitude of the factor loadings, intercepts, variances and covariances may differ across groups [43]. This is the baseline model because we assumed the same pattern of fixed and free parameters across groups, but no equality constraints were imposed. Therefore, we can compare it with more restrictive models. To estimate this model, the means of adherence were fixed to zero in all groups, but factor loadings, intercepts/thresholds, factor and residual variances were free to vary across groups.

2) **Metric, weak factorial or factor loading invariance**

This level of invariance requires that in addition to the constructs being measured by the same indicators as in configural invariance, factor loadings of those indicators must be equivalent across multiple groups [42]. A factor loading is the strength of the linear relation between a latent factor and each of the associated indicators [43, 82]. Under this type of invariance, factor loadings are constrained to be equal across groups, but no other equality constraints are imposed. Differences in this model imply differences across groups in how the latent variable affects its indicators [43]. Factor means cannot be compared across groups because of differences in the origin of the scale [43]. To estimate this model, means of adherence were fixed to zero in all groups, factor loadings were constrained to be equal, and the other parameters were freely estimated across groups.

3) **Scalar, strong factorial or intercept invariance**

This level of invariance justifies across time and between group comparisons [42]. It builds upon weak factorial, factor loading or metric invariance by requiring that the indicator intercepts also be equivalent across multiple groups [42]. An intercept is the origin or starting value of the adherence scale that the factor is based upon. To assess strong factorial invariance, factor loadings and intercepts were constrained to be equal across groups. This condition is also called “scalar invariance.” This level of invariance is required to make valid comparisons of means of latent factors across groups [43]. The model implies that any differences in means of the indicators are attributable to a difference in means of the latent variable. Thus, differences in covariances among indicators and in means of indicators are attributable to group differences in covariances and means on latent variables [82]. Under this invariance model, factor means for adherence were fixed in one group and free in the others, factor loadings and intercepts/thresholds and factor variances were constrained to be equal, whereas residual variances were freely estimated.

4) **Strict factorial or residual invariance**

This is the final criterion of invariance testing [42, 43]. There are two levels of strict factorial or residual invariance. The first level is invariance of factor variances, which represents overall error in prediction of the latent construct. The second level is invariance in error term estimates of individual indicator variables. Strict invariance testing assesses whether residual errors are equivalent across multiple groups. A residual is defined as uniqueness or measurement error associated with each measured indicator. This type of invariance extends strong factorial invariance, such that, residual variances are also constrained to be equal, in addition to factor loadings, intercepts and factor variances. Under this type of invariance, group differences are solely due to group differences in latent variables. This type of invariance likely does not hold in practice, because residual variances would vary from group to group even if all the groups were from one population. Therefore, we did not estimate models for this type of invariance.

## Abbreviations

ACTG: AIDS Clinical Trials Group
AIDS: Acquired immunodeficiency syndrome
ARMS: Adherence to Refills and Medication Scale
ART: Antiretroviral therapy
BMQ: Brief Medication Questionnaire
CD4: CD4 T lymphocytes
CFA: Confirmatory factor analysis
CFI: Comparative fit indices
CPCRA: Community Programs for Clinical Research on AIDS
DF: Degrees of freedom
EMP: Electronic medication packaging
HIV: Human immunodeficiency virus
IRB: Institutional Review Board
MAM: Medical Adherence Measure
MAQ: Morisky Adherence Questionnaire
MARS: Medication Adherence Report Scale
MAS: Medication Adherence Scale
MEASURE Evaluation: (Monitoring and Evaluation to ASsess and Use REsults) Evaluation
MedMaIDE: Medication Management Instrument for Deficiencies in the Elderly
MEMS: Medication event monitoring systems
MI: Measurement invariance
MOS: Medical Outcomes Study
RMSEA: Root mean square error of approximation
RNA: Ribonucleic acid
S.E.: Standard error
SERAD: Self-Reported Adherence
SMAQ: Simplified medication adherence questionnaire
SRSI: Self-Rating Scale Item
TLI: Tucker-Lewis indices
UNAIDS: United Nations AIDS Program
UNC: University of North Carolina
VAS: Visual Analog Scale
WHO: World Health Organization
WLSMV: Robust weighted least squares

## References

[1] UNAIDS (2019). FACT SHEET – GLOBAL AIDS UPDATE 2019: 2018 GLOBAL HIV STATISTICS.

[2] UNAIDS (2019). UNAIDS Data 2019.

[3] Sethi AK, Celentano DD, Gange SJ, Moore RD, Gallant JE (2003). Association between Adherence to Antiretroviral Therapy and Human Immunodeficiency Virus Drug Resistance. Clin Infect Dis - HIV/AIDS.

[4] Wood E, Hogg R, Yip B, Harrigan P, O'Shaughnessy M, Montaner J (2003). Effect of medication adherence on survival of HIV-infected adults who start highly active antiretroviral therapy when the CD4+ cell count is 0.200 to 0.350 x 10(9) cells/L. Ann Intern Med.

[5] Rai S, Mahapatra B, Raj SSPY, Venkatesh S, Shaukat M, Rewari BB (2013). Adherence to Antiretroviral Therapy and Its Effect on Survival of HIV-Infected Individuals in Jharkhand, India. PLoS One.

[6] Ayalew MB (2017). Mortality and Its Predictors among HIV Infected Patients Taking Antiretroviral Treatment in Ethiopia: A Systematic Review. AIDS Res Treat.

[7] WHO (2003). Adherence to long-term therapies project: evidence for action.

[8] Cohen MS, Chen YQ, McCauley M, Gamble T, Hosseinipour MC, Kumarasamy N, Hakim JG, Kumwenda J, Grinsztejn B, Pilott JH, Godbole SV, Mehendale S, Chariyalertsak S (2011). Prevention of HIV-1 Infection with Early Antiretroviral Therapy. N Engl J Med.

[9] UNAIDS (2014). 90-90-90: An ambitious treatment target to help end the AIDS epidemic.

[10] Stover J, Bollinger L, Izazola J, Loures L, DeLay P, Ghys P, F. T. m. w. group (2016). What Is Required to End the AIDS Epidemic as a Public Health Threat by 2030? The Cost and Impact of the Fast-Track Approach. PLoS One.

[11] Bezabhe W, Peterson G, Bereznicki L, Chalmers L, Gee P (2013). Adherence to antiretroviral drug therapy in adult patients who are HIV-positive in Northwest Ethiopia: a study protocol. BMJ.

[12] Mills EJ, Nachega JB, Buchan I, Orbinski J, Attaran A, Singh S, Rachlis B, Wu P, Cooper C, Thabane L, Wilson K, Guyatt GH, Bangsberg DR (2006). Adherence to Antiretroviral Therapy in Sub-Saharan Africa and North America A Meta-analysis. JAM.

[13] Molla AA, Gelagay AA, Mekonnen HS, Teshome DF (2018). Adherence to antiretroviral therapy and associated factors among HIV positive adults attending care and treatment in University of Gondar Referral Hospital, Northwest Ethiopia. BMC Infect Dis.

[14] Lam WY, Fresco P. Medication Adherence Measures: An Overview. Biomed Res Int. 2015. 10.1155/2015/217047. 10.1155/2015/217047 PMC4619779 26539470

[15] Stirratt MJ, Dunbar-Jacob J, Crane HM, Simoni JM, Czajkowski S, Hilliard ME, Aikens JE, Hunter CM, Velligan DI, Huntley K, Ogedegbe G, Rand CS, Schron E, Nilsen WJ (2015). Self-report measures of medication adherence behavior: recommendations on optimal use. Transl Behav Med.

[16] Simoni JM, Kurth AE, Pearson CR, Merrill JO, Frick PA, D. W. P (2006). Self-Report Measures of Antiretroviral Therapy Adherence: A Review with Recommendations for HIV Research and Clinical Management. AIDS Behav.

[17] Deyno S, Toma A (2014). Adherence to Antiretroviral Therapy in HIV-Positive Patients in Ethiopia: Review. J Trop Dis Public Health.

[18] Biressaw S, Abegaz WE, Abebe M, Taye WA, Belay M. Adherence to Antiretroviral Therapy and associated factors among HIV infected children in Ethiopia: unannounced home-based pill count versus caregivers’ report. BMC Pediatr. 2013;132(2013). 10.1186/1471-2431-13-132. 10.1186/1471-2431-13-132 PMC3766076 24229394

[19] Amberbir A, Woldemichael K, Getachew S, Girma B, Deribe K (2008). Predictors of adherence to antiretroviral therapy among HIV-infected persons: a prospective study in Southwest Ethiopia. BMC Public Health.

[20] Markos E, Worku A, Davey G (2008). Self-reports may overstate ART adherence [adherence to ART in PLWHA at Yirgalem Hospital, South Ethiopia]. J Health Dev.

[21] Knobel H, Alonso J, Casado JL, Collazos J, Gonzalez J, Ruiz I, Kindelan JM, Carmona A, Juegai J, A. O. o. b. o. t. G. S. Group (2002). Validation of a simpli®ed medication adherence questionnaire in a large cohort of HIV-infected patients: the GEEMA Study. AIDS.

[22] Degroote S, Vogelaers D, Vermeir P, Mariman A, Rick AD, Gucht BVD, Pelgrom J, Wanzeele FV, Verhofstede C, Vancauwenberghe J, Vandijck D (2014). Determinants of adherence in a cohort of Belgian HIV patients: a pilot study. Acta Clin Belg.

[23] Mao L, Buchanan A, Wong HTH, Persson A. Beyond mere pill taking: SMS reminders for HIV treatment adherence delivered to mobile phones of clients in a community support network in Australia. Health Soc Care Commun. 2018. 10.1111/hsc.12544. 10.1111/hsc.12544 29336111

[24] Malbergier A, Cardoso LD, R. A. d. Amaral (2014). Alcohol dependence and CD4 cell count: is there a relationship?. AIDS Care.

[25] Wang X, Wu Z (2007). Factors associated with adherence to antiretroviral therapy among HIV/AIDS patients in rural China. AIDS.

[26] Henry C, Pavese P, Blanc M, Labarère J, Leclercq P, Brion J-P. HIV infection and diabetes: Experience and quality of life in patients with two chronic diseases. Presse Med. 40(n° 10):e463–70, 2011 (octobre 2011). 10.1016/j.lpm.2011.05.019. 10.1016/j.lpm.2011.05.019 21831573

[27] V. Alikari, V. Matziou, M. Tsironi, N. Kollia, P. Theofilou, A. Aroni, E. Fradelos and S. Zyga, A modified version of the Greek Simplified Medication Adherence Questionnaire for hemodialysis patients, Health Psychol Res. 5:6647, 2017, 10.4081/hpr.2017.6647. 10.4081/hpr.2017.6647 PMC5452632 28603780

[28] Awori V, Mativo P, Yonga G, Shah R (2018). The association between asymptomatic and mild neurocognitive impairment and adherence to antiretroviral therapy among people living with human immunodeficiency virus. S Afr J HIV Med.

[29] Cantú-Rodríguez OG, Sánchez-Cárdenas M, Gutiérrez-Aguirre CH, Jaime-Pérez JC, Mancias-Guerra C, González-Llano O, Gómez-Almaguer D (2014). Cultural factors related to adherence to imatinib in CML: A Mexican perspective. Hematology.

[30] Pacífico J, Gutiérrez C (2015). Rev Peru Med Exp Salud Publica.

[31] Platt MO, Evans D, Keegan PM, McNamara L, Parker IK, Roberts LM, Caulk AW, Seifu D, Amogne W, Penny C, R. L. G. Jr (2016). Low cost method to monitor patient adherence to HIV antiretroviral therapy using multiplex cathepsin zymography. Mol Biotechnol.

[32] Suárez FO, Plumed JS, Valentín MP, Palomo PP, Cepeda MM, Aguiar DL, G. d. E. Vatren (2011). Validation on the simplified medication adherence questionnaire (SMAQ) in renal transplant patients on tacrolimus. Nefrología.

[33] Toll BA, Martin SAMDJ, Jatlow P, O’Malley SS (2007). Factor structure and validity of the Medication Adherence Questionnaire (MAQ) with cigarette smokers trying to quit. Nicotine Tob Res.

[34] Brar A, Babakhani A, Salifu M, Jindal R (2014). Evaluation of Non-adherence in Patients Undergoing Dialysis and Kidney Transplantation practice patterns survey. Transplatation Proceedings.

[35] Abheiden C, Reuler AV, Fuijkschot W, Vries JD, Thijs A, Boer MD (2016). Aspirin adherence during high-risk pregnancies, a questionnaire study. Pregnancy Hypertens.

[36] Ossareh S, Tabrizian S, Zebarjadi M, Joodat RS (2014). Prevalence of Depression in Maintenance Hemodialysis Patients and Its Correlation With Adherence to Medications. Iran J Kidney Dis.

[37] Barraco A, Rossi A, Nicolo G (2012). Description of Study Population and Analysis of Factors Influencing Adherence in the Observational Italian Study “Evaluation of Pharmacotherapy Adherence in Bipolar Disorder” (EPHAR). CNS Neurosci Therapeutics.

[38] Ganasegeran K, Rashid A (2017). The prevalence of medication nonadherence in post-myocardial infarction survivors and its perceived barriers and psychological correlates: a cross-sectional study in a cardiac health facility in Malaysia. Patient Preference Adherence.

[39] Lalić J, Veličković-Radovanović R, Mitić B, Paunović G, Cvetković T (2014). Immunosuppressive Medication Adherence in Kidney Transplant Patients. Med Princ Pract.

[40] He Y, Tan EH, Wong ALA, Tan CC, Wong P, Lee SC, Tai BC (2018). Improving medication adherence with adjuvant aromatase inhibitor in women with breast cancer: study protocol of a randomised controlled trial to evaluate the effect of short message service (SMS) reminder. BMC Cancer.

[41] Rosa VG, Nicolás FG, Moreno RG, Romero MMV, Carvajal MTM, Pérez YRG (2013). Adherence and toxicity to tyrosine kinase inhibitor. Farm Hosp.

[42] S. Bialosiewicz, K. Murphy and T. Berry, "Do our Measures Measure up? The Critical Role of Measurement Invariance," in An Introduction to Measurement Invariance Testing: Resource Packet for Participants , Claremont, 2013.

[43] Sousa KH, West SG, Moser SE, Harris JA, Cook SW (2012). Establishing Measurement Invariance: English and Spanish Paediatric Asthma Quality of Life Questionnaire. Nurs Res.

[44] Wicherts JM. The importance of measurement invariance in neurocognitive ability testing. Clin Neuropsychol. 2016. 10.1080/13854046.2016.1205136. 10.1080/13854046.2016.1205136 27356958

[45] Vandenberg RJ, Lance CE (2000). A review and synthesis of the measurement invariance literature: suggestions, practices, and recommendations for organizational research. Organ Res Methods.

[46] Thomas J, Reynolds H, Bevc C, Tsegaye A (2014). Integration opportunities for HIV and family planning services in Addis Ababa, Ethiopia: an organizational network analysis. BMC Health Serv Res.

[47] Thomas J, Reynolds H, Alterescu X, Bevc C, Tsegaye A. Improving referrals and integrating family planning and HIV services through organizational network strengthening. Health Policy Plan. 2016:302–8. 10.1093/heapol/cz058. 10.1093/heapol/czv058 PMC6296328 26135363

[48] Gadkari AS, McHorney CA (2012). Unintentional non-adherence to chronic prescription medications: How unintentional is it really?. BMC Health Serv Res.

[49] Peterson RA (1994). A Meta-analysis of Cronbach's Alpha. Oxford Univ Press J Consumer Res.

[50] Streiner DL (2003). Starting at the Beginning: An Introduction to Coefficient Alpha anCOEFFSITCRIEINNTE ARLPdHA Internal Consistency. J Pers Assess.

[51] Clark LA, Watson D (1995). Constructing Validity: Basic Issues in Objective Scale Development. Am Psychol Assoc Psychol Assess.

[52] Mislevy JL, Rupp AA (2012). "Concurrent Validity," in Encyclopedia of Research Design.

[53] Balasubramanian CK (2015). The Community Balance and Mobility Scale Alleviates the Ceiling Effects Observed in the Currently Used Gait and Balance Assessments for the Community-Dwelling Older Adults. Geriatr Physical Ther.

[54] Portney LG, Watkins MP (2015). Foundations of clinical research: Applications to practice.

[55] Piland SG, Motl RW, Ferrara MS, Peterson CL (2003). Evidence for the Factorial and Construct Validity of a Self-Report Concussion Symptoms Scale. J Athl Train.

[56] West S, Finch J, Curran P, Hoyle R (1995). Structural equation models with nonnormal variables: Problems and remedies. Structural equation modeling: Concepts, issues, and applications.

[57] Muthén L, Muthén B (1998). Mplus User’s Guide, Seventh Edition ed.

[58] StataCorp., Stata Statistical Software: Release 12.. College Station: StataCorp LP. 2011.

[59] Dunlow N, Phillips C, Broder HL (2007). Concurrent validity of the COHIP. Commun Dent Oral Epidemiol.

[60] Lifson AR, Demissie W, Tadesse A, Ketema K, May R, Yakob B, Metekia M, Slater L, Shenie T (2012). Barriers to Retention in Care as Perceived by Persons Living with HIV in Rural Ethiopia: Focus Group Results and Recommended Strategies. J Int Assoc Providers AIDS Care.

[61] Morisky DE, Ang A, Krousel-Wood M, Ward HJ (2008). Predictive Validity of a Medication Adherence Measure in an Outpatient Setting. J Clin Hypertens.

[62] Chesney M, Ickovics JR, Chambers D, Gifford A, Neidig J, Zwickl B, A. W. &. P. C. C. &. A. W. G. O. T. O. C. O. T. A. A. C. T. G. (AACTG) (2000). Self-reported adherence to antiretroviral medications among participants in HIV clinical trials: The AACTG Adherence Instruments. AIDS Care.

[63] Morisky DE, Green LW, Levine DM. Concurrent and Predictive Validity of a Self-Reported Measure of Medication Adherence and Long-Term Predictive Validity of Blood Pressure Control. Med Care. 1986;24(1). 10.1097/00005650-198601000-00007 3945130

[64] Nguyen T-M-U, Caze AL, Cottrell N (2013). What are validated self-report adherence scales really measuring?: a systematic review. Br J Clin Pharmacol.

[65] Bader A, Kremer H, Erlich-Trungenberger, Rojas R, Lohmann M, Deobald O, Lochmann R, Altmeyer P, Brockmeyer N (2006). An adherence typology: coping, quality of life, and physical symptoms of people living with HIV/AIDS and their adherence to antiretroviral treatment. Med Sci Monit.

[66] Luszczynska A, Sarkar Y, Knoll N (2007). Received social support, self-effi cacy, and fi nding benefi ts in disease as predictors of physical functioning and adherence to antiretroviral therapy. Patient Educ Couns.

[67] Mannheimer S, Matts J, Telzak E, Chesney M, Child C, Wu A, G. F. G, T. B. C. P. f. C. R. o. AIDS (2005). Quality of life in HIV-infected individuals receiving antiretroviral therapy is related to adherence. AIDS Care.

[68] Parsons T, Braaten A, Hall C, Robertson K (2006). Better quality of life with neuropsychological improvement on Haart. Health Qual Life Outcomes.

[69] Ruiz-Pérez I, Olry de Labry-Lima A, López-Ruz M, del Arco-Jiménez A, Rodríguez-Baño J, Causse-Prados M, Pasquau-Liaño J, Martín-Rico P, Prada-Pardal J, de la Torre-Lima J, López-Gómez M, Marcos M, Muñoz N, Morales D, Muñoz I (2005). Clinical status, adherence to HAART and quality of life in HIV-infected patients receiving antiretroviral treatment. Enferm Infecc Microbiol Clin.

[70] S. Sahay, K. S. Reddy and S. Dhayarkar, "Optimizing adherence to antiretroviral therapy," The Indian Journal of Medical Research, pp. 134(6):835-849, https://www.ncbi.nlm.nih.gov/pmc/articles/PMC3284093/, 2011. 10.4103/0971-5916.92629 PMC3284093 22310817

[71] Robbins RN, Spector AY, Mellins CA, Remien RH (2014). Optimizing ART Adherence: Update for HIV Treatment and Prevention. Curr HIV/AIDS Rep.

[72] McNairy ML, El-Sadr WM (2014). Antiretroviral Therapy for the Prevention of HIV Transmission: What Will It Take?. Clin Infect Dis.

[73] Haberer JE, Sabin L, Amico KR, Galárraga COO, Tsai AC, Vreeman RC, Wilson I, Sam-Agudu NA, Blaschke TF, Vrijens B, Mellins CA, Remien RH, Weiser SD, Elizabeth (2017). Improving antiretroviral therapy adherence in resource-limited settings at scale: a discussion of interventions and recommendations. J Int AIDS Soc.

[74] WHO, "Global Health Observatory (GHO) data HIV/AIDS," WHO, 17 May 2019. [Online]. Available: https://www.who.int/gho/hiv/en/. [Accessed 17 May 2019].

[75] Mora P, Berkowitz A, Contrada R, Wisnivesky J, Horne R, Leventhal H, Halm E (2011). Factor structure and longitudinal invariance of the Medical Adherence Report Scale-Asthma. Psychol Health.

[76] Kim ES, Yoon M, Wen Y, Luo W, Kwok O-m (2015). Within-Level Group Factorial Invariance With Multilevel Data: Multilevel Factor Mixture and Multilevel MIMIC Models. Struct Equation Modeling A Multidiscip J.

[77] Vrijens B, Geest SD, Hughes DA, Przemyslaw K, Demonceau J, Ruppar T, Dobbels F, Fargher E, Morrison V, Lewek P, Matyjaszczyk M, Mshelia C, Clyne W, Aronson JK, Urquhart J (2012). A new taxonomy for describing and defining adherence to medications. Br J Clin Pharmacol.

[78] Pilcher CD, Nguyen SAFTQ, Foust E, Wolf L, Williams D, Ashby R, O’Dowd JO, McPherson JT, Stalzer B, Hightow L, Miller WC, Eron JJ, Cohen MS, Leone PA (2005). Detection of Acute Infections during HIV Testing in North Carolina. N Engl J Med.

[79] Stramer SL, Glynn SA, Kleinman SH, Strong DM, Caglioti S, Wright DJ, Dodd RY, Busch MP (2004). Detection of HIV-1 and HCV Infections among Antibody-Negative Blood Donors by Nucleic Acid–Amplification Testing. N Engl J Med.

[80] WHO (2006). Scaling up antiretroviral therapy in resource-limited settings: treatment guidelines for a public health approach.

[81] Kool MB, García IL-C, Mewes R, Silva JAPD, Vangronsveld K, Wismeijer AAJ, Lumley MA, Middendorp HV, Bijlsma JWJ, Crombez G, Rief W, Geenen R, R. v. d. Schoot (2014). Measurement invariance of the Illness Invalidation Inventory (3*I) across language, rheumatic disease and gender. BMJ Clin Epidemiol Res.

[82] Bollen K (1989). Structural equations with latent variables.

[83] Widaman KF, Reise S (1997). "Exploring the measurement invariance of psychological instruments: Applications in the substance use domain," in The science of prevention.

